# Complete Genome Sequence of Staphylococcus arlettae Strain P2, Isolated from a Laboratory Environment

**DOI:** 10.1128/MRA.00696-19

**Published:** 2019-11-07

**Authors:** Hiroki Yu, Makoto Taniguchi, Kazuma Uesaka, Apirak Wiseschart, Kusol Pootanakit, Yudai Nishitani, Yota Murakami, Koichiro Ishimori, Kentaro Miyazaki, Kei Kitahara

**Affiliations:** a Graduate School of Chemical Sciences and Engineering, Hokkaido University, Sapporo, Hokkaido, Japan; b Ambitious Leader’s Program Fostering Future Leaders to Open New Frontiers in Materials Science (ALP), Hokkaido University, Sapporo, Hokkaido, Japan; c Oral Microbiome Center, Taniguchi Dental Clinic, Takamatsu, Kagawa, Japan; d Graduate School of Bioagricultural Sciences, Nagoya University, Nagoya, Aichi, Japan; e Institute of Molecular Biosciences, Mahidol University, Salaya, Nakhon Pathom, Thailand; f Department of Life Science and Biotechnology, Bioproduction Research Institute, National Institute of Advanced Industrial Science and Technology, Tsukuba, Ibaraki, Japan; g Department of Chemistry, Faculty of Science, Hokkaido University, Sapporo, Hokkaido, Japan; h Department of Computational Biology and Medical Sciences, Graduate School of Frontier Sciences, The University of Tokyo, Kashiwa, Chiba, Japan; University of Rochester School of Medicine and Dentistry

## Abstract

Staphylococcus arlettae is one coagulase-negative species in the bacterial genus *Staphylococcus*. Here, we describe the closed complete genome sequence of *S. arlettae* strain P2, which was obtained using a hybrid approach combining Oxford Nanopore long-read and Illumina MiSeq short-read sequencing data.

## ANNOUNCEMENT

Staphylococcus arlettae is a species of the genus *Staphylococcus* and was reportedly first isolated from skin or nares of poultry or goats (1). *S. arlettae* has since been additionally isolated from veterinary (2–6) and clinical (7–10) samples. Other *S. arlettae* isolation sites include soil (11), cell phone surfaces (12), and a disused biological safety cabinet (13).

The *S. arlettae* strain selected for whole-genome sequencing reported here was isolated from a biological laboratory in a university in Sapporo, Japan. Microbes on the laboratory floor surface were swabbed using a sterilized moistened swab. The swab was used to streak an LB plate, and one colony, which appeared on the plate after incubation overnight at 37°C, was purified by colony streaking onto a fresh LB plate. The procedure was performed thrice. Analysis of the 16S rRNA gene of the resulting isolate (named P2) revealed that it shared 99.9% identity with the 16S rRNA sequence of *S. arlettae* strain CVD059 (9). Although draft genome sequences of *S. arlettae* strains, including CVD059, have already been reported by some groups (2, 14), none have been shown as closed sequences, motivating us to determine the first closed genome of an *S. arlettae* strain.

Before DNA extraction, strain P2 was inoculated in LB broth, and cells were cultured at 37°C until early stationary phase (the doubling time of P2 was approximately 38 min). High-molecular-weight genomic DNA was prepared from a harvested bacterial pellet using the MagAttract high-molecular-weight (HMW) DNA kit (Qiagen) according to the manufacturer’s instructions. The obtained genomic DNA was subjected to long-read and short-read sequencing at the Oral Microbiome Center at Taniguchi Dental Clinic in Japan. Default parameters were used for all software, unless otherwise specified.

Long-read sequencing was performed using a GridION X5 system (Oxford Nanopore Technologies [ONT]); 1.0-μg unfragmented genomic DNA was used for library construction using a ligation sequencing kit (ONT). The prepared library was applied to a FLO-MIN106 R9.41 flow cell (ONT). The long-read sequences, which were base called using Guppy v.3.0.3 (ONT), generated 59,502 reads (756 Mb) with an average length of 12,711 bp during a 10-h runtime (numbers are those for reads after quality filtering, with an average Phred quality value of >8.0 using NanoFilt v.2.3.0 [15]; the raw data contained 104,000 reads, with an average length of 8,957 bp).

For short-read sequencing, the paired-end (2 × 156-bp) Nextera DNA library (prepared using Nextera DNA Flex library prep kit [Illumina]) was sequenced on a MiSeq instrument. Raw sequencing data were processed using the FASTQ preprocessing program fastp v.0.19.5 (16) for the purpose of trimming adapters and low-quality data, yielding 1.05 million short reads with an average length of 152.8 bp.

For complete *de novo* genome assembly, both long-read and short-read data were processed using Unicycler v.0.4.4 (17), followed by a final polishing step using Pilon v.1.23 (18), generating a single circular contig for the chromosome with a length of 2,629,900 bp (G+C content of 33.7%) and another circular contig for a plasmid with a length of 22,364 bp (G+C content of 30.1%). To confirm that both circular contigs have no structural misassembly, we used the software program SV-Quest (K. Uesaka, unpublished data), which maps the short-read sequences back to the two contigs, detecting no signals for structural gaps and other inconsistencies. Automatic annotation was then performed using the annotation pipeline DFAST v.1.1.0 (19), provided by DDBJ, which predicted 2,550 coding sequences as well as 22 rRNA genes and 60 tRNA genes. Compared with CVD059, P2 had a chromosome that was 45 kbp shorter, which showed a symmetrical identity of 93.8% and gapped identity of 99.2%. CVD059 is reported to have 2,439 coding sequences (9). There has been no report that CVD059 contains a plasmid.

To our knowledge, this represents the first closed genome sequence report for an *S. arlettae* strain registered to a public database, providing an essential basis for detailed comparative analysis of *S. arlettae* genomes in the future.

### Data availability.

The closed complete chromosomal and plasmid sequences were deposited at DDBJ/EMBL/GenBank under accession numbers AP019698 and AP019699, respectively. The versions described in the manuscript are the first versions, AP019698.1 and AP019699.1, respectively. Raw sequencing data were deposited in the DDBJ SRA database under the accession numbers DRX167894 and DRX167895.
